# Tuina ameliorates sleep disturbances in PCPA-treated rats through Piezo1-mediated calcium signaling

**DOI:** 10.3389/fphar.2026.1777810

**Published:** 2026-03-26

**Authors:** Yaqi Ding, Yan Xuan, Tingting Liang, Mengjie Zheng, Jinying Gao

**Affiliations:** 1 Department of Central Laboratory, Ruijin Hospital, Luwan Branch, Shanghai Jiao Tong University School of Medicine, Shanghai, China; 2 Department of Endocrinology, Ruijin Hospital Luwan Branch, Shanghai Jiao Tong University School of Medicine, Shanghai, China; 3 TCM of Department, Jiading Branch of Shanghai General Hospital, Shanghai Jiao Tong University School of Medicine, Shanghai, China; 4 Department of Tuina, Shanghai Municipal Hospital of Traditional Chinese Medicine, Shanghai University of Traditional Chinese Medicine, Shanghai, China; 5 Department of Traditional Chinese Medicine, Ruijin Hospital Luwan Branch, Shanghai Jiao Tong University School of Medicine, Shanghai, China

**Keywords:** calcium signaling, PCPA-treated rats, Piezo1, sleep disturbances, tuina

## Abstract

**Objectives:**

Insomnia is a prevalent sleep disorder that severely impairs physical and mental health. Clinical research has shown that Tuina, a form of therapeutic massage, can improve sleep quality and restore normal sleep patterns, yet the underlying biological mechanism remains unclear. This study aimed to determine whether Tuina improves sleep-related behaviors by modulating the Piezo1–calcium signaling pathway through mechanical stimulation of the nervous system.

**Materials and methods:**

A rat model of sleep disturbances was induced using PCPA. Rats received daily Tuina on the dorsal regions corresponding to the Governor Vessel and Bladder meridians. sleep-related behavioral parameters, cognitive function, hippocampal neurotransmitters, and expression of Piezo1 and downstream proteins (CaM, CaN) were assessed. The Piezo1 agonist Yoda1 was used to verify mechanistic involvement.

**Results:**

Tuina treatment significantly improved sleep-related behavioral parameters and cognitive performance in PCPA-treated rats. At the molecular level, PCPA-induced sleep disruption led to Piezo1 upregulation, intracellular calcium overload, and subsequent overactivation of the CaM/CaN signaling pathway. These alterations were accompanied by neurotransmitter imbalance and hippocampal neuronal damage. Tuina intervention effectively suppressed Piezo1 expression, normalized calcium homeostasis, and inhibited downstream CaM/CaN activation, thereby restoring neurotransmitter levels and preserving neuronal integrity. Critically, the therapeutic benefits of Tuina were largely reversed by co-administration of the Piezo1 agonist Yoda1, supporting the involvement of Piezo1-mediated calcium signaling in its mechanism of action.

**Conclusion:**

Tuina’s therapeutic effect involves suppression of Piezo1-dependent calcium signaling, contributing to normalization of hippocampal function and neurotransmitter balance. This provides mechanistic evidence that Tuina’s mechanical stimulation modulates sleep-related behavioral endpoints via central mechanosensitive ion channels.

## 1 Introduction

Insomnia is a prevalent sleep disorder that severely impairs cognitive function, emotional stability, and overall quality of life. Epidemiological data indicate that approximately one-third of adults experience transient or chronic insomnia, with its incidence rising in modern societies because of increased psychosocial stress and altered circadian rhythms (Tian-Ji et al., 2023; Eric et al., 2024; Marta et al., 2022). Persistent sleep loss is closely associated with neurological dysfunction, neuroinflammation, and impaired neurotransmission in brain regions such as the hippocampus and prefrontal cortex (Francesca et al., 2020; Zi-Xuan et al., 2024). Current pharmacological treatments, including benzodiazepines and melatonin receptor agonists, can effectively promote sleep onset; however, long-term administration often results in tolerance, dependence, and cognitive side effects (Jing-Li et al., 2023). Consequently, there is growing interest in exploring non-pharmacological approaches with higher safety and sustained efficacy.

Tuina, a major branch of traditional Chinese medicine, is a manual therapy that employs rhythmic mechanical pressure, kneading and rolling techniques along specific meridians and acupoints to restore physiological balance. Modern clinical studies have demonstrated that Tuina significantly improves subjective sleep quality and normalises the circadian rhythm in patients with insomnia (Sai et al., 2020; Efthalia et al., 2020; Felipe Rodrigues et al., 2018). Compared with pharmacological sedatives, Tuina shows advantages in safety and sustainability, with minimal side effects and holistic regulation of neural, endocrine and immune systems. Preclinical evidence suggests that abdominal Tuina may ameliorate insomnia by modulating the hypothalamic CRH/CRHR1 pathway within the brain-gut axis (Ye et al., 2021), and upregulates neurotrophic factors such as brain-derived neurotrophic factor (BDNF) and insulin-like growth factor-1 (IGF-1) (Rong et al., 2024). Furthermore, Tuina has been reported to attenuate depressive-like behaviour in chronic stress models through activation of the IGF-1/Nrf2 antioxidant pathway, indicating that mechanical stimulation can elicit complex molecular responses in neural tissues (Xingxing et al., 2025).

Recent advances in mechanobiology have elucidated how mechanical forces generated during Tuina are transduced into biochemical signals. Central to this process are mechanosensitive ion channels, with Piezo1 serving as a key molecular sensor that converts membrane deformation into Ca^2+^ influx and downstream intracellular responses (Lai et al., 2025). Structurally suited to detect changes in membrane tension, Piezo1 is widely expressed in neurons, astrocytes, and vascular endothelial cells, where it regulates critical functions such as neuronal excitability, neurovascular coupling, and synaptic plasticity (Andrea et al., 2024; Osama et al., 2022).

Notably, Piezo1 exhibits adaptive changes under pathological conditions such as sleep disruption. For instance, Zhang et al. reported significant upregulation of Piezo1 in the hippocampus following sleep deprivation, correlating with heightened neuronal excitability (Zi-Qing et al., 2023). Further supporting its role in sleep-related pathology, Piezo1 inhibition in the rat basal forebrain was shown to reverse fear memory deficits induced by sleep deprivation (Tao et al., 2021). Moreover, the expression of Piezo1 is subject to circadian regulation, and disruption of this rhythm—leading to elevated Piezo1 activity during the sleep phase—has been linked to conditions such as nocturia and sleep disruption (Tatsuya et al., 2017). Together, these findings position Piezo1 as a functionally significant mediator in the mechanobiological mechanisms underlying Tuina, with potential relevance to sleep disorders.

Delving into the molecular mechanisms, the calcium signaling transduction initiated by Piezo1 channel activation represents the critical link connecting mechanical stimulation to neural function regulation. Intracellular calcium ions, as important second messengers, regulate neurotransmitter release, gene expression, and synaptic plasticity through spatiotemporal concentration changes (Das et al., 2008). Calcium signaling plays a vital role in regulating sleep-wake cycles (Fengfei et al., 2016). Calcineurin (CaN), as a calmodulin-dependent phosphatase, is considered a molecular switch in sleep regulation. Recent research shows that CaN regulates the phosphorylation state of synaptic proteins by balancing protein kinase A (PKA) activity, thereby influencing sleep homeostasis (Yimeng et al., 2024). However, this delicate balance can be easily disrupted under pathological conditions. Sustained calcium overload leads to excessive CaN activation, triggering a series of pathological processes including synaptic dysfunction, neuroinflammatory responses, and neuronal apoptosis (Pallab et al., 2025; Jiaxing et al., 2024).

Based on this evidence, we hypothesized that Tuina improves sleep disturbances through mechanical regulation of Piezo1 channels, leading to restoration of Ca^2+^ homeostasis and inhibition of downstream CaN overactivation. To test this hypothesis, a p-chlorophenylalanine (PCPA)-induced rat model of sleep disturbances was established to mimic serotonin depletion. Tuina intervention was applied to the dorsal regions corresponding to the Governor Vessel and Bladder meridians, while the Piezo1 agonist Yoda1 was used to assess mechanistic dependence. By integrating behavioural, neurochemical, and histopathological analyses, along with molecular assessment of Piezo1–Ca^2+^–CaN signalling, this study aimed to elucidate the mechanobiological mechanism by which Tuina alleviates sleep disturbances and to provide a theoretical basis for its clinical application as a safe and effective therapeutic approach.

## 2 Materials and Methods

### 2.1 Animals and experimental design

Male Sprague–Dawley rats (200–220 g) were obtained from Shanghai SLAC Laboratory Animal Co., Ltd. (Shanghai, China) and housed under standard laboratory conditions (22 °C ± 2 °C, 50% ± 5% humidity, 12 h light/dark cycle) with *ad libitum* access to food and water. All animal experiments were performed according to procedures approved by the Institutional Animal Care and Use Committee of our University and conformed to the NIH Guide for the Care and Use of Laboratory Animals (2011). Sleep disturbances was induced by intraperitoneal injection of PCPA (Sigma–Aldrich, St. Louis, MO, United States; 400 mg/kg/day) for two consecutive days, as described previously (Yuchen et al., 2024; Wing-Yan et al., 2025), followed by a repeated administration on day 7 to consolidate modelling. After completion of the first-stage modelling, rats in the Tuina and Tuina + Yoda1 groups received daily intervention for 21 days. The Piezo1 agonist Yoda1 (10 μM/kg; MedChemExpress, Monmouth Junction, NJ, United States) was administered intraperitoneally 30 min before Tuina treatment.

### 2.2 Tuina intervention procedure

The Tuina procedure was performed once daily by two professionally trained practitioners. Stimulation targeted the dorsal Governor Vessel (GV) and bilateral Bladder meridian (BL) regions corresponding to T6–L6 spinal segments (China Association of Acupuncture and Moxibustion, 2025). Animals were gently restrained in the prone position without anesthesia. Unidirectional linear pushing from the vertex to the coccygeal region was applied along GV and BL meridians (20 strokes per meridian; ∼2 s per stroke; velocity 2–3 cm/s; ∼30 strokes/min), with an applied pressure maintained at 3.8–4.2 N. Total duration was approximately 4 min. Sequential rhythmic pressure (1 Hz) was applied to GV20 (Baihui), GV14 (Dazhui), BL15 (Xinshu), BL17 (Geshu), BL20 (Pishu), BL23 (Shenshu), and GV6 (Jizhong) for 30 ± 2 s per acupoint using the same pressure range (3.8–4.2 N). A ∼10-s transition interval separated the two procedures. Total daily stimulation time was maintained at 9–10 min. Contact force and stimulation duration were continuously monitored using the FingerTPS system (Pressure Profile Systems, Los Angeles, CA, United States), and pressure–time curves were recorded to ensure stimulation stability and reproducibility. Control animals were handled identically without mechanical stimulation.

### 2.3 Sleep behavior assessment

Sleep-related behavior was evaluated using the pentobarbital sodium–induced sleep test. Following intraperitoneal injection of pentobarbital sodium (40 mg/kg; Sinopharm Chemical Reagent Co., Shanghai, China), the sleep latency (time from injection to loss of righting reflex) and sleep duration (time from loss to recovery of righting reflex) were recorded. The tests were performed on day 3 (model validation) and day 21 (post-treatment).

### 2.4 Morris water maze test

Spatial learning and memory performance were assessed using the Morris water maze (MWM). The apparatus consisted of a circular pool (diameter 160 cm) filled with opaque water maintained at 24 °C ± 1 °C. Rats underwent four training trials per day for five consecutive days, during which they learned to locate a hidden platform submerged 2 cm below the water surface. On the sixth day, a probe trial was conducted by removing the platform to evaluate memory retention. Parameters including escape latency, path length, target quadrant residence time, and swimming trajectory were recorded using an automated tracking system.

### 2.5 Determination of neurotransmitter levels

The hippocampal levels of γ-aminobutyric acid (GABA), dopamine (DA), serotonin (5-HT), norepinephrine (NE), 5-hydroxyindoleacetic acid (5-HIAA), and glutamate (Glu) were determined using commercially available ELISA kits (Jiangsu Meimian Industrial Co., Ltd., Yancheng, China) according to the manufacturer’s instructions with minor modifications. Briefly, freshly isolated hippocampal tissues were weighed and homogenised in ten volumes (w/v) of ice-cold phosphate-buffered saline (PBS, 0.01 M, pH 7.4) using a glass–Teflon homogeniser (IKA, Germany). The homogenates were centrifuged at 12,000 × g for 15 min at 4 °C (Eppendorf 5430R, Hamburg, Germany), and the supernatants were collected for analysis. For each assay, 100 μL of standard or sample was added to pre-coated wells and incubated for 60 min at 37 °C, followed by washing and incubation with biotinylated antibody and HRP-streptavidin conjugate. After addition of the TMB substrate and termination with 2 M H_2_SO_4_, absorbance was measured at 450 nm using a BioTek Synergy HTX microplate reader (Agilent Technologies, United States). Concentrations of neurotransmitters were calculated from the standard curve fitted with a four-parameter logistic equation and expressed as ng or nmol per mg of total protein. Total protein concentration was measured using a BCA kit (Beyotime, Shanghai, China) for normalisation.

### 2.6 Western blot analysis

Hippocampal tissues were homogenised in RIPA lysis buffer (Beyotime, Shanghai, China) containing protease and phosphatase inhibitors (Thermo Fisher Scientific, United States). Protein concentrations were measured using a BCA kit (Beyotime, Shanghai, China). Equal amounts of protein (30 µg) were separated on 10% SDS–PAGE gels (Bio-Rad, Hercules, CA, United States) and transferred onto PVDF membranes (Millipore, Billerica, MA, United States). Membranes were blocked with 5% non-fat milk (Sangon Biotech, Shanghai, China) for 1 h and incubated overnight at 4 °C with primary antibodies: Piezo1 (Abcam, United Kingdom; Cat. No. ab128245), CaM (CST, United States; Cat. No. 4856S), CaN (1:1000, CST, United States; Cat. No. 2614S), GAPDH (1:5000, Proteintech, Wuhan, China; Cat. No. 60004-1-Ig). After incubation with HRP-conjugated secondary antibodies (Beyotime, Shanghai, China), bands were visualised using an ECL detection kit (Thermo Fisher Scientific, United States).

### 2.7 Immunohistochemistry

After behavioural testing, rats were perfused with 0.9% saline followed by 4% paraformaldehyde (Solarbio, Beijing, China). Brains were post-fixed overnight, embedded in paraffin and sectioned at 4 µm. After deparaffinisation, antigen retrieval was performed in citrate buffer (pH 6.0; Servicebio, Wuhan, China) using a microwave oven for 10 min. Endogenous peroxidase was blocked with 3% H_2_O_2_, and sections were blocked in 5% BSA (Solarbio, Beijing, China) for 30 min. Primary antibodies used were: Piezo1 (Abcam, United Kingdom; ab128245), CaN (CST, United States; 2614S). Sections were incubated overnight at 4 °C, followed by HRP-conjugated secondary antibody (Servicebio, Wuhan, China) for 1 h at 37 °C. DAB (Servicebio, Wuhan, China) was used for visualisation, and sections were counterstained with haematoxylin. Images were captured under an Olympus BX53 microscope (Olympus Corporation, Tokyo, Japan), and staining intensity (average optical density, AOD) was quantified using ImageJ.

### 2.8 Histopathological examination

Tissues were fixed in 4% paraformaldehyde, embedded in paraffin, and sectioned for hematoxylin–eosin (H&E) and Nissl staining (Beyotime, Shanghai, China). Morphological features of hippocampal neurons, including cell density, nuclear integrity, and Nissl body distribution, were examined under light microscopy to assess structural integrity and neuronal viability.

### 2.9 Measurement of intracellular calcium concentration

The intracellular calcium ion concentration in hippocampal tissue was determined using the fluorescent probe Fura-2/AM (Invitrogen, Thermo Fisher Scientific, United States), following a modified method described previously (Yao et al., 2017). Briefly, freshly isolated hippocampal tissues were minced and homogenised in ice-cold HEPES buffer (132 mM NaCl, 3 mM KCl, 10 mM glucose, 10 mM HEPES, and 2 mM CaCl_2_, pH 7.4) to prepare a single-cell suspension (1 × 10^6^ cells/mL). The suspension was filtered through a 40 μm nylon mesh (BD Falcon, United States) to remove debris and undissociated tissue. Cell viability was assessed by trypan blue exclusion (0.4%; Thermo Fisher Scientific, United States) and consistently exceeded 90% in all samples. Cell density was adjusted to 1 × 10^6^ viable cells/mL in HEPES buffer.

The suspension was incubated with 5 μM Fura-2/AM at 37 °C for 45 min in a 5% CO_2_ incubator, then washed three times with Ca^2+^-free HEPES buffer to remove excess dye. Fluorescence intensity was measured using a Shimadzu RF-5300 fluorescence spectrophotometer (Kyoto, Japan) with excitation at 340 and 380 nm and emission at 510 nm. The ratio (R = F_340_/F_380_) was recorded and used to calculate the intracellular calcium concentration according to the equation: *[Ca2+]= Kd (R-Rmin)/(RmaxRmin)*, where *K*_*d*_ = 224 nM is the dissociation constant of Fura-2, *R*_max_ is obtained after adding 10% Triton X-100, and *R*_min_ is obtained after adding 5 mM EGTA. Background autofluorescence was subtracted from all measurements using unloaded cell suspensions. And each sample was analyzed in five technical replicates, with the mean value used for statistical analysis. Results are expressed as mean ± SD in nM [Ca^2+^]i.

### 2.10 Statistical analysis

All data are expressed as mean ± SD. Statistical analysis was performed using GraphPad Prism 8.0 (GraphPad Software, San Diego, CA, United States). For the Morris water maze escape latency during training days and body weight, two-way repeated measures ANOVA (group × day) was used. Sphericity was assessed using Mauchly’s test, and Greenhouse–Geisser corrections were applied when the sphericity assumption was violated. Post hoc multiple comparisons were performed using Tukey’s test. For all other comparisons, differences between groups were analyzed by one-way ANOVA followed by Tukey’s *post hoc* test. All behavioral assessments and quantifications were performed under blinded conditions: experimenters conducting the pentobarbital-induced sleep test, Morris water maze test, and all subsequent molecular and histological analyses were unaware of group allocation. Data entry and statistical analyses were also performed by researchers blinded to the experimental groups. A value of p < 0.05 was considered statistically significant.

## 3 Results

### 3.1 Tuina ameliorated behavioral deficits in rats with PCPA-induced sleep disturbances

**FIGURE 1:**
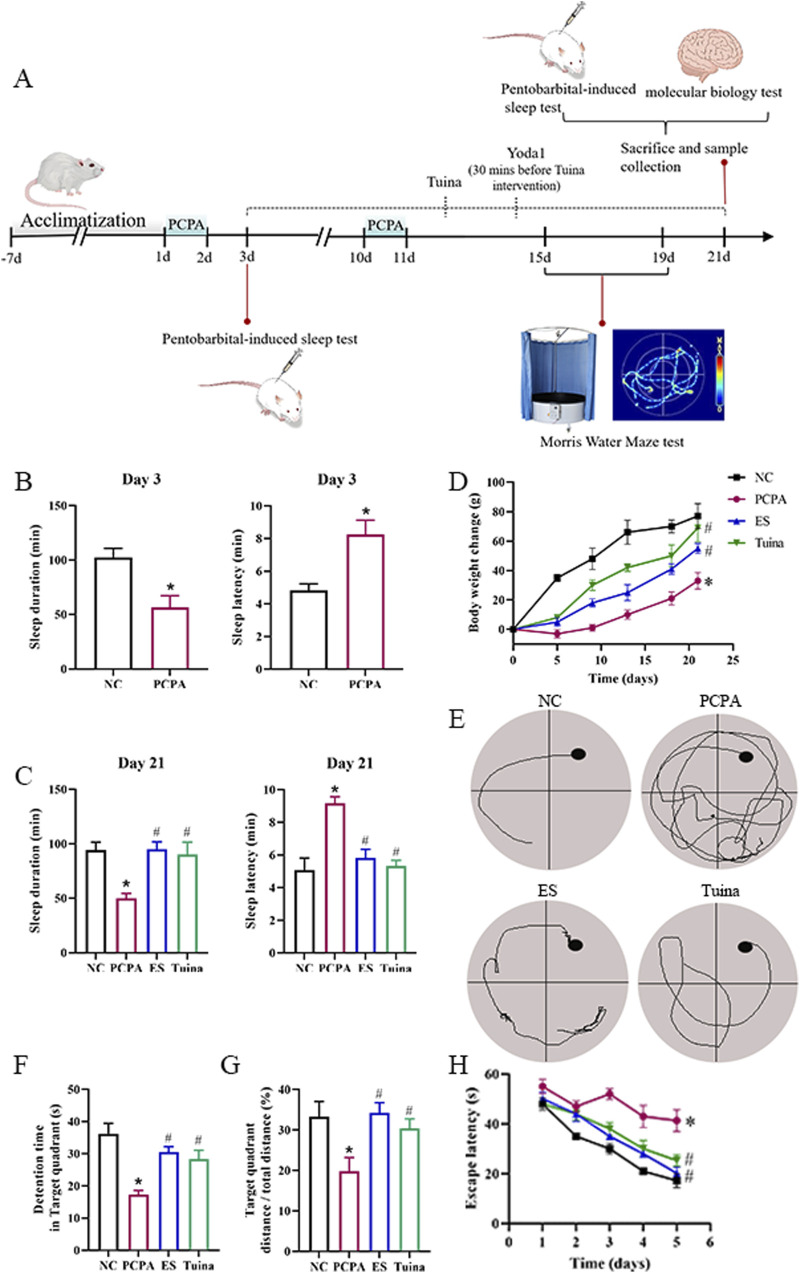
Tuina ameliorated behavioral deficits in rats with PCPA-induced sleep disturbances. **(A)** Schematic representation of the experimental design. **(B)** Comparisons of sleep latency and sleep duration between the NC and PCPA groups on day 3. **(C)** Sleep latency and sleep duration on day 21 post-treatment. **(D)** Changes in body weight over the study period. **(E)** Sample swim paths from the Morris Water Maze test. **(F–H)** Behavioral metrics were evaluated during the Morris water maze testing phase. n = 5. *p < 0.05 vs. NC, #p < 0.05 vs. PCPA.

As illustrated in Figure 1A, sleep disturbances was induced by intraperitoneal injection of PCPA, followed by repeated PCPA administration after 7 days to reinforce model stability. Beginning on the day after completion of the first-stage modeling, rats received daily interventions for 21 days. To further elucidate the mechanistic involvement of Piezo1 in Tuina-mediated effects, a subgroup of rats received the Piezo1 agonist Yoda1 in combination with Tuina treatment. On the third day post-modeling, rats in the PCPA group exhibited markedly prolonged sleep latency and reduced sleep duration compared with those in the NC group (Figure 1B), confirming successful model establishment. After 21 days of intervention, both Tuina and ES treatments significantly shortened sleep latency and extended sleep duration relative to the PCPA group (Figure 1C), suggesting a restorative effect on sleep behavior. Moreover, PCPA-treated rats displayed attenuated weight gain throughout the experiment, whereas Tuina and ES interventions partially reversed this trend (Figure 1D). In the Morris water maze test, representative swimming trajectories demonstrated that Tuina-treated rats adopted more directed search paths resembling those of the NC and ES groups (Figure 1E). Quantitative analyses revealed that PCPA-induced sleep disturbances significantly decreased residence time in the target quadrant (Figure 1F) and the percentage of target quadrant distance (Figure 1G), while escape latency was markedly prolonged (Figure 1H) compared with the NC group. Both Tuina and ES interventions effectively reversed these impairments, as evidenced by longer target quadrant residence time, higher target distance percentage, and reduced escape latency. Collectively, these results demonstrate that Tuina effectively ameliorated behavioral and cognitive deficits induced by PCPA, improving sleep-related behavioral endpoints and learning–memory performance to a degree comparable with the pharmacological action of estazolam.

### 3.2 Tuina alleviates PCPA-induced hippocampal cell damage and neurotransmitter dysregulation

**FIGURE 2:**
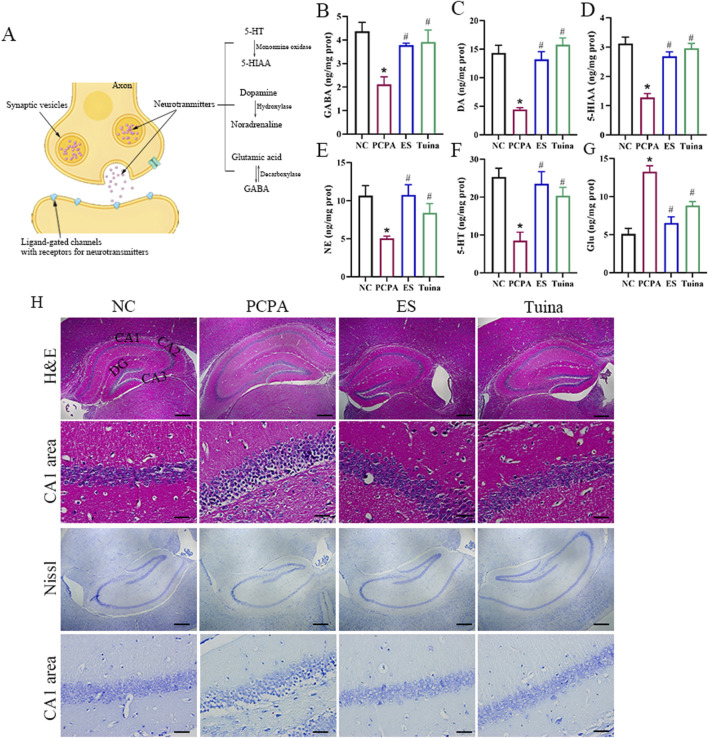
Tuina alleviates PCPA-induced hippocampal cell damage and neurotransmitter dysregulation. **(A)** Diagram outlining neurotransmitter-related pathways. **(B–G)** Hippocampal levels of GABA **(B)**, DA **(C)**, 5-HIAA **(D)**, NE **(E)**, 5-HT **(F)**, and Glu **(G)**. **(H)** H&E and Nissl staining of the hippocampal region. For each group (NC, PCPA, ES, Tuina), low-magnification overviews (scale bar = 100 μm) and higher-magnification views (scale bar = 10 μm) are shown. n = 5 in each group. *p < 0.05 vs. NC, #p < 0.05 vs. PCPA.

To evaluate the impact of Tuina on PCPA-induced sleep disturbances and associated neurological disruptions, we assessed changes in key neurotransmitter levels and hippocampal histopathology. Neurotransmitter synthesis, release, and receptor interactions are critically involved in synaptic signaling and neural function (Figure 2A). In the PCPA-treated rats, significant dysregulation of multiple neurotransmitters was observed. Specifically, levels of 5-HT, 5-HIAA, NE, DA, and GABA were markedly decreased in the PCPA group compared to the NC group (Figures 2E,F), whereas Glu levels were significantly elevated (Figure 2G). These alterations indicate a disruption in monoaminergic and inhibitory/excitatory neurotransmitter balance following sleep disruption. Notably, Tuina treatment significantly reversed these abnormalities, restoring 5-HT, NE, DA, GABA, and 5-HIAA levels toward normal, while reducing the elevated Glu concentration. Histological examination of the hippocampus further supported these findings. H&E and Nissl staining (Figure 2H) revealed that PCPA administration induced significant neuronal damage, including shrunken nuclei and reduced Nissl bodies, indicative of impaired neuronal integrity and metabolic activity. In contrast, Tuina treatment effectively attenuated these pathological changes, preserving neuronal structure and enhancing Nissl body density, comparable to the protective effects observed in the ES group. Collectively, these results demonstrate that Tuina alleviates PCPA-induced neurotransmitter dysregulation and hippocampal cellular injury.

### 3.3 Tuina downregulates Piezo1 expression in the hippocampus of PCPA-treated rats

**FIGURE 3:**
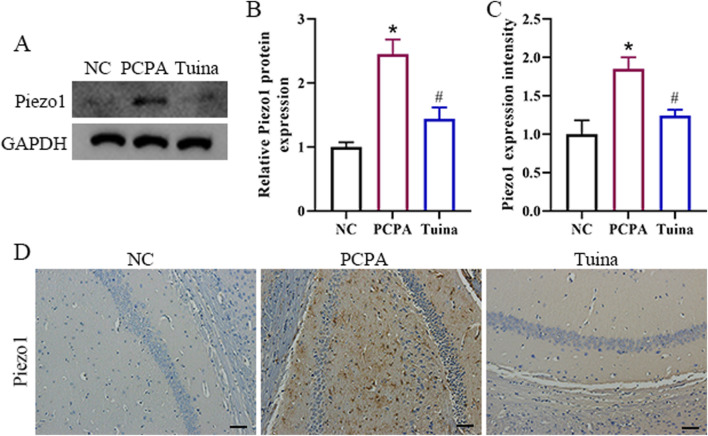
Tuina downregulates Piezo1 expression in the hippocampus of PCPA-treated rats. **(A)** Western blot analysis for Piezo1 expression. **(B)** Quantitative analysis of Piezo1 protein expression normalized to GAPDH. **(C)** Quantitative analysis of Piezo1 Immunohistochemistry intensity. **(D)** Representative Immunohistochemistry images of Piezo1 expression. Scale bar = 50 μm. n = 5 in each group; the data are presented as the mean ± SD. *p < 0.05 vs. NC, #p < 0.05 vs. PCPA.

To investigate whether the mechanosensitive ion channel Piezo1 contributes to the therapeutic mechanisms of Tuina, we systematically evaluated its expression in the hippocampus. Western blot analysis demonstrated that PCPA administration significantly elevated Piezo1 protein expression compared to the NC group, with quantitative assessment revealing an approximate 2.3-fold increase. Notably, Tuina treatment effectively counteracted this upregulation, restoring Piezo1 protein levels to near baseline values (Figures 3A,B). Immunohistochemistry analysis provided additional confirmation, showing substantially intensified Piezo1 signals distributed across hippocampal regions in PCPA-treated rats. Importantly, Tuina intervention significantly attenuated Piezo1 immunoreactivity, reducing signal intensity to patterns comparable with control conditions (Figures 3C,D). This observation can be explained by remote mechano-neural regulatory mechanisms: peripheral mechanical stimulation activates spinal-brainstem-thalamic-cortical and limbic pathways (Pengfei et al., 2018; Liu et al., 2022) while simultaneously modulating autonomic nervous activity and systemic inflammatory status (Liu et al., 2025; Li et al., 2022; Tong et al., 2024), collectively reshaping the hippocampal microenvironment—including Ca^2+^ homeostasis, neuroinflammatory balance, and glial cell activation—to drive adaptive changes in Piezo1 expression. Together, these findings establish that sleep disruption induced by PCPA triggers substantial Piezo1 overexpression in hippocampal formations, and that Tuina effectively normalizes this dysregulation.

### 3.4 Therapeutic effect of Tuina on PCPA-induced sleep disturbances involves Piezo1-mediated signaling

**FIGURE 4:**
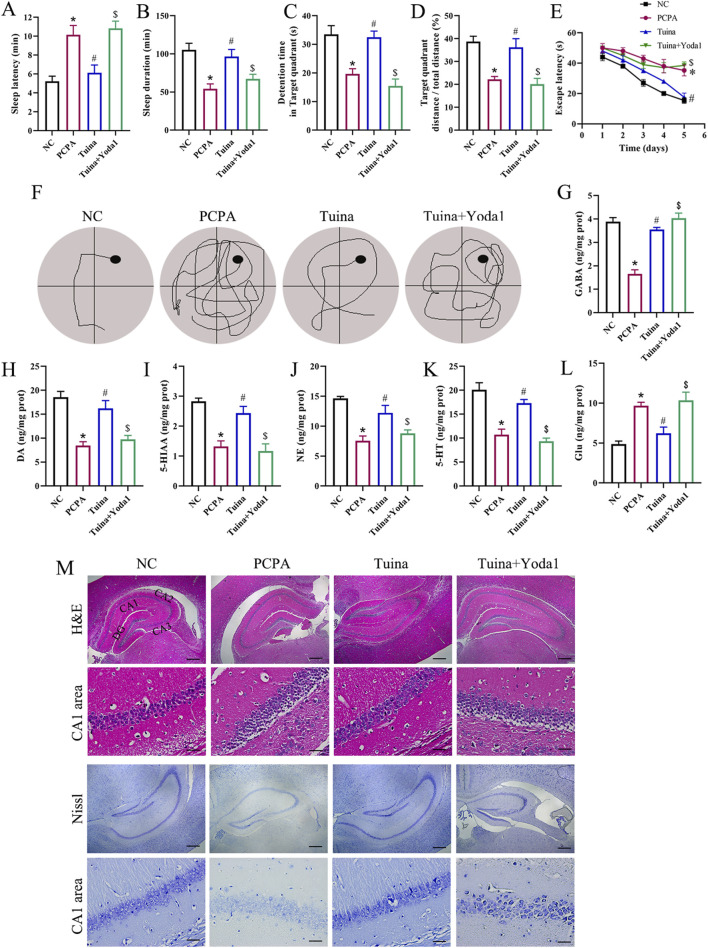
Therapeutic effect of Tuina on PCPA-induced sleep disturbances involves Piezo1-mediated signaling. **(A,B)** Sleep latency and sleep duration of rats in different groups. **(C–E)** Assessment of behavioral parameters. **(G–L)** The levels of GABA, DA, 5-HIAA, NE, 5-HT, and Glu. **(M)** H&E and Nissl staining of the hippocampal region. For each group (NC, PCPA, Tuina, Tuina + Yoda1), low-magnification overviews (scale bar = 100 μm) and higher-magnification views (scale bar = 10 μm) are shown. n = 5 in each group; the data are presented as the mean ± SD. *p < 0.05 vs. NC, #p < 0.05 vs. PCPA, $p < 0.05 vs. Tuina.

To explore the therapeutic efficacy of Tuina and its potential reliance on Piezo1 signaling, we examined the influence of the Piezo1 agonist Yoda1 on behavioral, neurochemical, and histopathological parameters in PCPA-treated rats. Tuina markedly enhanced sleep-related behavioral endpoints, as evidenced by shortened sleep latency and prolonged sleep duration (Figures 4A,B). These improvements were significantly diminished by Yoda1 co-administration, suggesting that Piezo1 activation mitigates the beneficial effects of Tuina. In the Morris water maze, Tuina-treated rats displayed superior spatial learning and memory performance, while concurrent Yoda1 exposure weakened these cognitive gains (Figures 4C–F). Neurochemical profiling further revealed that Tuina re-established neurotransmitter homeostasis, elevating GABA, DA, 5-HT, NE, and 5-HIAA levels and lowering Glu concentrations; this regulatory effect was partially suppressed by Yoda1 (Figures 4G–L). Histological observations supported these findings, showing that Tuina preserved hippocampal neuronal integrity and Nissl body structure, whereas Yoda1 co-treatment induced mild neuronal atrophy and cytoplasmic disorganization (Figure 4M). Overall, these results demonstrate that Tuina effectively counteracts sleep-related behavioral endpoints, neurochemical, and neuronal abnormalities through a mechanism involving Piezo1-mediated mechanosensory signaling, and that Piezo1 activation compromises its therapeutic efficacy.

### 3.5 Tuina ameliorates sleep disturbances in PCPA-treated rats by a mechanism involving Piezo1-mediated calcium signaling

**FIGURE 5:**
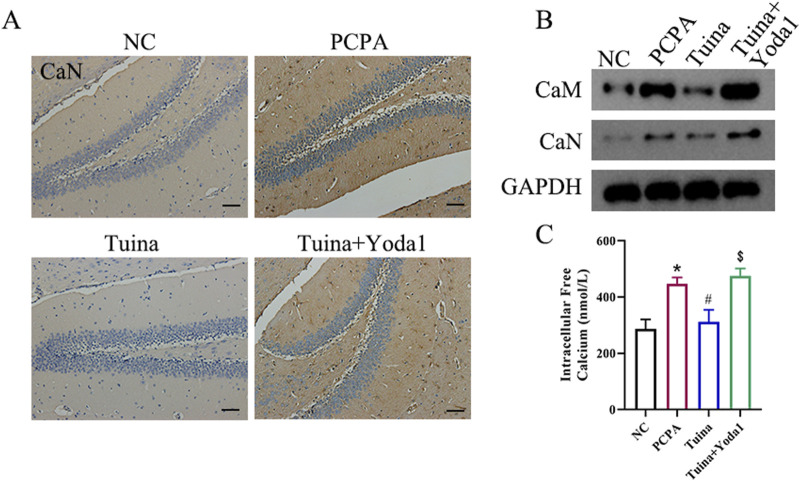
Tuina ameliorates sleep disturbances in PCPA-treated rats by a mechanism involving Piezo1-mediated calcium signaling. **(A)** Immunohistochemical detection for CaN. Scale bar = 50 μm. **(B)** Western blot analysis for CaM and CaN expression. **(C)** Intracellular calcium concentration measured by Fura-2/AM in mixed hippocampal cell suspensions. n = 5 in each group; the data are presented as the mean ± SD. *p < 0.05 vs. NC, #p < 0.05 vs. PCPA, $p < 0.05 vs. Tuina.

We next examined hippocampal calcium signaling to determine the role of Piezo1 in Tuina’s therapeutic effect. Immunohistochemical staining revealed that CaN expression was markedly reduced in Tuina-treated rats compared with the PCPA group, indicating that Tuina attenuates calcium-dependent signaling activity (Figure 5A). This downregulation was significantly reversed by co-administration of Yoda1. Consistently, Western blot analysis demonstrated that Tuina decreased the expression of both CaM and CaN proteins, while Yoda1 co-treatment restored their levels toward those observed in PCPA rats (Figure 5B). Furthermore, Tuina significantly lowered intracellular Ca^2+^ concentration in mixed hippocampal cell suspensions, and this reduction was partially reversed by Yoda1 exposure (Figure 5C). Taken together, these results indicate that Tuina mitigates sleep disruption-associated alterations by suppressing Piezo1-mediated calcium influx and downstream CaM/CaN signaling, whereas activation of Piezo1 by Yoda1 reverses these inhibitory effects, suggesting that the Piezo1-Ca^2+^ axis contributes to the therapeutic mechanism of Tuina.

## 4 Discussion

Insomnia represents a significant health burden, affecting cognitive function, emotional stability, and overall quality of life. While clinical trials have demonstrated that Tuina significantly improves Pittsburgh Sleep Quality Index (PSQI) scores and restores normal sleep architecture in patients with insomnia (Zhen et al., 2024; Yangshen et al., 2022), the biological mechanisms have remained elusive. This knowledge gap has limited the widespread acceptance and optimization of Tuina in modern sleep medicine. The present study addresses this gap by investigating a novel mechanistic hypothesis: that Tuina improves sleep-related behavior through regulation of the Piezo1-calcium signaling pathway. This focus on mechanosensitive ion channels represents an innovative approach to understanding how mechanical stimulation translates into neurological effects. The Piezo1 channel was selected as a promising candidate due to its established role as a primary mechanotransducer in various biological systems and its presence in neural tissues.

The PCPA-induced sleep disturbance model was selected based on its well-established mechanism of serotonin depletion through irreversible inhibition of tryptophan hydroxylase (Yanpo et al., 2022). This model reliably reproduces key features of human insomnia, including prolonged sleep latency, fragmented sleep patterns, and cognitive impairments (Wenhui et al., 2023). Our validation data showing significant sleep disturbances on day 3 post-PCPA administration aligns with previous reports (Dongge et al., 2024), confirming the model’s reliability for investigating sleep-related mechanisms. The specificity of PCPA’s action on the serotonergic system makes it particularly suitable for studying interventions that might modulate multiple neurotransmitter systems.

The selection of the GV and BL regions for Tuina intervention in this study was based on established clinical acupoint principles, drawing from both traditional Chinese medicine theory and contemporary clinical research evidence. As demonstrated in the systematic review by Lu et al., Shenmen (HT7), Sanyinjiao (SP6), Baihui (GV20), Zusanli (ST36), and Neiguan (PC6) represent the most frequently utilized acupoints for treating senile insomnia, with the BL and GV identified as the primary meridians (Geling et al., 2024). Our Tuina protocol aligns remarkably well with these findings, specifically targeting the dorsal GV and BL regions, including key acupoints such as BL15 and BL20. Notably, while acupuncture typically employs discrete point stimulation, our Tuina intervention adopted a more comprehensive mechanical approach by manipulating extended segments of these meridians. This methodological distinction may account for certain unique aspects of our findings. The mechanical pressure applied during Tuina likely activates a broader array of mechanoreceptors and stimulates more extensive neural pathways compared to localized needle insertion. This broader stimulation pattern may be particularly effective in modulating the distributed neural networks involved in sleep-wake regulation, potentially contributing to the observed therapeutic effects through enhanced neuromodulation of sleep-related circuits.

Tuina’s effectiveness in reducing sleep latency and extending sleep duration aligns with clinical observations (Zhen et al., 2024), while its positive effects on Morris water maze performance demonstrate broader cognitive benefits. These findings extend previous reports by showing that mechanical stimulation can produce improvements comparable to pharmacological interventions like estazolam, yet through distinct mechanisms. The protective effects of Tuina on hippocampal neurons complement growing evidence that sleep disturbances cause structural brain changes (Zichen et al., 2023; Shu-Jie et al., 2023). Our observation that Tuina preserved neuronal architecture and Nissl body density suggests it may counteract the neurodegenerative processes associated with chronic sleep loss. The restoration of 5-HT and 5-HIAA levels following Tuina intervention provides mechanistic insight into how manual therapy might normalize serotonergic function. This is particularly relevant given the established role of 5-HT in sleep-wake regulation and the effectiveness of serotonergic drugs in treating insomnia (Jaime, 2011). The rebalancing of multiple neurotransmitter systems, including GABA, glutamate, and monoamines, suggests that Tuina acts as a neuromodulator with broad-spectrum effects. The normalization of the glutamate-GABA balance is crucial given the role of excitatory-inhibitory imbalance in sleep disorders (Xiao-Hua et al., 2023). This multi-target approach may explain Tuina’s clinical efficacy while avoiding the side effects associated with single-target pharmacological agents.

The Piezo1 channel has emerged as a principal mechanosensor in various biological systems, capable of converting diverse mechanical stimuli into electrochemical signals. Previous studies have demonstrated that different types of mechanical forces can differentially regulate Piezo1 expression and activity. For instance, in vascular endothelial cells, laminar shear stress downregulates Piezo1 expression through KLF2-mediated transcription, thereby protecting against calcium overload-induced apoptosis (Ahalya et al., 2025). Conversely, pathological mechanical stimuli such as high-magnitude cyclic stretch typically upregulate Piezo1 expression, leading to sustained Ca^2+^ influx and cellular dysfunction (Yang et al., 2021). Recent research identifies a novel chronic traumatic encephalopathy mechanism: fluid shear stress disrupts Ca^2+^ homeostasis in depolarized neurons via Piezo1-mediated Ca^2+^ influx, triggering aberrant vesicle release and apoptosis (Tianyu et al., 2025). In contrast to these established patterns of mechanical regulation, our findings demonstrate that Tuina induces downregulation of Piezo1 expression, rather than the upregulation typically associated with pathological mechanical stress. This bidirectional regulatory capacity suggests that the biological effects of mechanical stimulation may depend on specific parameters including force magnitude, frequency, duration, and pattern. The functional significance of Piezo1 downregulation was unequivocally demonstrated through Yoda1 co-administration experiments. Yoda1, a specific Piezo1 agonist that stabilizes the channel’s open state, significantly attenuated Tuina’s therapeutic benefits across behavioral, neurochemical, and histological parameters. This pharmacological rescue experiment provides evidence that Piezo1 inhibition is involved in Tuina’s effects on sleep-related behavioral endpoints.

An important consideration arising from our findings is that Tuina, as a form of somatic mechanical stimulation, was not directly applied to the hippocampal region, yet significant changes in Piezo1 expression were observed in the hippocampus. This apparent spatial disconnect necessitates an interpretation grounded in remote mechano-neural regulatory mechanisms. It is well established that mechanical stimulation of the body surface does not exert its effects via direct physical conduction to deep brain structures, but rather through activation of peripheral mechanoreceptors, including Aβ and C-fiber-associated mechanoreceptors in the skin and deep tissues. These afferent signals are relayed via spinal-brainstem-thalamic-cortical and limbic pathways, ultimately modulating central nervous system function and inducing molecular plasticity in distant brain regions (Pengfei et al., 2018; Liu et al., 2022). Accordingly, the observed alterations in hippocampal Piezo1 expression and downstream Ca^2+^-dependent signaling likely reflect indirect regulation through changes in neural circuit activity, neurotransmitter release, and neuroinflammatory status, rather than a direct mechanical effect on hippocampal tissue. Moreover, Tuina has been shown to modulate autonomic nervous tone, hypothalamic-pituitary-adrenal (HPA) axis activity, and systemic inflammatory responses (Liu et al., 2025; Li et al., 2022; Tong et al., 2024). These systemic regulatory effects can further reshape the hippocampal microenvironment, including Ca^2+^ homeostasis, glial cell activation, and local neuroinflammatory balance, thereby driving adaptive changes in mechanosensitive channel expression.

The hippocampus represents a novel context for Piezo1 research due to its established role in sleep-dependent memory consolidation and vulnerability to sleep deprivation (Lisa et al., 2023). The presence and regulability of the channel in this region suggest that mechanosensitive mechanisms may contribute to previously unrecognized aspects of hippocampal function in sleep regulation. Furthermore, the relationship between Piezo1 and other mechanosensitive channels warrants consideration. Although our study specifically focused on Piezo1, it is plausible that Tuina’s mechanical stimulation simultaneously modulates other mechanosensitive elements, including Piezo2 (Yinggang et al., 2024), TRPV4 (Na et al., 2020), and stretch-activated potassium channels (Melania et al., 2023). The coordinated regulation of multiple mechanosensing systems could produce the integrated physiological responses observed following Tuina intervention. Future studies employing more comprehensive mechanochannel profiling could elucidate these potential interactions.

The observed normalization of hippocampal intracellular Ca^2+^ levels and CaM/CaN expression following Tuina intervention provides a coherent pathway linking mechanical stimulation to neuronal excitability regulation. Calcium signaling has emerged as a crucial regulator of sleep-wake cycles. Astrocytic Ca^2+^ signaling is reduced during sleep and is involved in the regulation of slow wave sleep (Laura et al., 2020), with CaN recently identified as a sleep-promoting phosphatase (Xin et al., 2025). Our findings suggest that Tuina fine-tunes this system, preventing both insufficient signaling and pathological overactivation. This balanced modulation may explain Tuina’s ability to improve sleep-related behavioral endpoints without causing excessive sedation.

Several limitations should be acknowledged. First, the pentobarbital-induced sleep test primarily reflects sedative sensitivity rather than directly measuring natural sleep architecture. Future studies incorporating EEG/EMG recordings are necessary to comprehensively evaluate sleep structure. Second, while handling and restraint were strictly matched across groups, the absence of a sham Tuina control limits definitive separation of specific mechanical effects from non-specific tactile stimulation. Third, although the Yoda1 rescue experiment provides pharmacological evidence for Piezo1 necessity, definitive causal proof requires complementary approaches such as Piezo1-specific inhibitors or genetic knockout models, which are now underway in our laboratory. Fourth, our Ca^2+^ measurements and Piezo1 expression analyses were performed on bulk hippocampal tissue and cannot distinguish contributions from specific cell types (neurons vs. glia) or subpopulations. Future studies using cell-type-specific calcium imaging and immunofluorescence staining are required to resolve these issues. Despite these limitations, the convergent behavioral, molecular, and pharmacological evidence supports Piezo1 as a mediator of Tuina’s therapeutic effects on sleep disturbances.

Our study establishes a mechanistic framework connecting peripheral mechanical stimulation to central sleep regulation through Piezo1-Ca^2+^-CaM-CaN signaling. The integration of traditional acupoint selection with modern mechanobiology creates new opportunities for developing evidence-based manual therapies. Future research should explore several promising directions: First, the optimal stimulation parameters for Piezo1 modulation need systematic investigation to refine treatment protocols. Second, studies examining Tuina’s effects in other sleep-related brain regions would provide a more comprehensive understanding of its central actions. Third, comparative effectiveness studies across different insomnia subtypes could identify which patients are most likely to benefit from Tuina intervention. Finally, the development of more selective Piezo1 modulators might lead to novel pharmacological approaches inspired by manual therapy mechanisms. In conclusion, our findings position Tuina as a legitimate mechanobiological intervention that targets specific molecular pathways to restore sleep-related behavioral endpoints homeostasis. The identification of Piezo1 as a mediator opens new avenues for both optimizing traditional manual therapies and developing novel mechano-based treatments for sleep disorders.

## Data Availability

The original contributions presented in the study are included in the article/supplementary material, further inquiries can be directed to the corresponding authors.
